# Design proposals for the preferred service ecosystem for senior citizens living at home: a service design study

**DOI:** 10.3389/fpubh.2025.1706153

**Published:** 2025-12-05

**Authors:** Christophe Eward Kattouw, Karina Aase, Petter Viksveen

**Affiliations:** 1 SHARE—Centre for Resilience in Healthcare, Faculty of Health Sciences, University of Stavanger, Stavanger, Norway; 2 Department of Public Health, Faculty of Health Sciences, University of Stavanger, Stavanger, Norway; 3 Department of Quality and Health Technology, Faculty of Health Sciences, University of Stavanger, Stavanger, Norway

**Keywords:** age-friendly services, senior citizens, service design, service ecosystem, timely home care, transportation, community health services, health services research

## Abstract

**Introduction:**

Significant gaps exist between the preferred and the existing service ecosystem for senior citizens living at home emphasizing the need for transformation. This study aimed to develop design proposals for the preferred service ecosystem for senior citizens living at home.

**Methods:**

Nine service design workshops were conducted with multiple stakeholder groups (*n* = 58), including senior citizens (aged 67 years or older), carers, health care professionals, municipal home care managers, municipal advisers, a bus driver, and representatives from the regional transportation provider. Stakeholders identified timely home care provision and age-friendly mobility as key areas for improvement. An inductive thematic analysis was applied to the workshop data.

**Results:**

Design proposals were developed including prototypes and design principles across seven domains: (1) enabling self-reliance; (2) housing and buildings; (3) urban mobility; (4) collaboration and education; (5) real-time communication; (6) resource organization and flexible scheduling; and (7) methods and tools to reshape patterns of thought. Design principles focused on enabling sufficient time and resources, improving accessibility, and changing mental models. These proposals may support senior citizens’ self-reliance, free up time and resources for service providers, and create time buffers for service delays.

**Discussion:**

The design proposals demonstrate considerable interconnectedness and transferability across timely home care provision and age-friendly mobility. They may improve predictability and adaptive capacity for both senior citizens and service providers.

## Introduction

To enhance dignity, participation and wellbeing in the context of a global aging population, communities should be transformed to enable senior citizens to live their lives according to what matters to them, while also serving as a resource for the wider community. Achieving this, calls for the development of a service ecosystem that includes age-friendly housing, mobility options, outdoor spaces, welcoming social venues, community support and needs-based health services (1–5).

A service ecosystem can be defined as “a relatively self-contained, self-adjusting system of resource-integrating actors connected by shared institutional arrangements and mutual value creation through service exchange” (6). The stakeholders within this ecosystem may have multiple perspectives on service delivery (7–9) and their experiences and expectations of the service delivery process are also emphasized, rather than outcomes alone (10–12).

There is growing awareness among governmental decision-makers and policymakers that global aging calls for a service ecosystem approach (13, 14). One example is the World Health Organization’s whole-system strategy for developing age-friendly cities and communities, highlighting the interconnectedness of domains such as home care services, public transport, and the built environment (15).

Despite ongoing requests from senior citizen representatives and policy makers, a considerable gap remains between the preferred and the existing service ecosystem. This includes services that compromise senior citizens’ independence and dignity by being task-focused rather than person-centered, fragmented instead of integrated, and lacking continuity. Additionally, inadequate public transportation and the absence of suitable venues for socializing may limit senior citizens’ social participation (16–20).

An idealized design approach applies systems thinking and stakeholder involvement, aiming to close the gaps between the preferred and the existing systems (21, 22). Service design complements this by incorporating a human-centric and co-creative mindset, using various toolsets and visualisations, and applying a service ecosystem approach (23–27).

Service design has been used to develop solutions in health care, such as alcohol prevention (28), medication administration (29), wait times for breast cancer exams (30), health care access for marginalized groups (31), and electronic health records (32).

However, no previous research applied an idealized design approach using service design methods to close the gaps between the existing and preferred service ecosystem for senior citizens living at home. Furthermore, the development of design proposals for service ecosystems for senior citizens has previously not been described. Therefore, the aim of this study is to develop design proposals for the preferred service ecosystem for senior citizens living at home.

## Materials and methods

### Study design

A qualitative service design study (25, 26, 33) was conducted to develop design proposals for the preferred service ecosystem for senior citizens living at home. The study used a workshop-based approach involving different stakeholder groups, including senior citizens (aged 67 years or older), carers, health care professionals, municipal home care managers, municipal advisers, a bus driver, and representatives from the regional transportation provider.

The workshops followed the three iterative phases of design thinking: define point of view, ideate, and prototype. Defining the point of view involves selecting a specific area for improvement to guide the ideation phase (34). Two improvement areas were selected based on previous input from the involved stakeholder groups: age-friendly mobility and the continuity of health services (35). Based on “How might we” questions, potential solutions were generated through brainstorming processes. The results then formed the foundation for developing prototypes (34). Prototypes or potential design solutions are detailed design proposals which address how things might be experienced and implemented differently in the future (24, 25, 33).

The study represents the third stage of a multi-phased idealized design project in a Norwegian municipality. The consolidated criteria for reporting qualitative research (COREQ) checklist were used to report this study (36).

### Setting, sample and recruitment

In Norway, most home care services and local public transport are governmental responsibilities. However, the national government delegates the provision of home care services to the country’s 357 municipalities and shares the responsibility for local public transport with these municipalities and the 15 counties. Municipalities and counties have the autonomy to organize public transport and home care services as they see fit, provided they comply with legal requirements (e.g., accessible, safe and sound care). Additional services, such as cleaning and attending senior citizens centers, may incur charges, home care services should be provided free of charge. Public transport, including buses and trams, typically involves a fee.

The current study was conducted in one of Norway’s 20 largest municipalities (>75,000 inhabitants), including both urban and rural areas. It offers home care and reablement services, whereas public transport is provided by the county and delegated to a regional transportation provider.

Representatives from a senior citizen organization were consulted in Workshop 1 and selected age-friendly mobility and timely home care provision as key priorities for developing design proposals. This initial selection laid the foundation in directing the focus towards the two improvement areas addressed in subsequent workshops. They emphasized close connection between these areas and their importance in supporting senior citizens’ self-reliance, activity, and social engagement. Age-friendly mobility was understood by them as including accessible infrastructures and predictable public transport. Timely home care was understood as aligned with senior citizens’ social needs, daily routines, and lifestyle preferences.

Participant recruitment was facilitated by municipal home care leaders. The first author recruited senior citizens representatives, municipal advisers and representatives from the regional transportation provider, who in turn recruited a bus driver. All participants received written information about the project and provided written informed consent. Three senior citizens were unable to attend workshops due to illness and or mobility issues. In total, 53 participants were involved in the workshops, including senior citizens, carers, health care professionals, managers, and transportation and municipal advisers (Table 1).

**Table 1:** Sample, workshop methods and findings.

Workshop	Participants (*n* = 53) ^a^	Methods ^b^	Findings
1	Senior citizen representatives (*n* = 5, age 72–79)	Visual presentation of the preferred service ecosystem. Brainstorming and discussion. (Define point of view; Ideate)	Agreement to improve timely home care provision and age-friendly mobility. 16 overall design solutions on age-friendly mobility (*n* = 11) and timely home care provision (*n* = 5).
Timely home care provision			
2	Home care managers including the head of the municipal home care services (*n* = 12)	Lateral thinking task to warm up creativity. Negative brainstorming (What can worsen timely home care provision?). Bodystorming: groups (*n* = 3) prepared for and simulated a selected quote on timeliness in 3 minutes. Think-pair- share + sticky notes on possible design solutions. (Ideate)	467 single design solutions divided into 30 sub-themes (such as technology, competence, and communication skills), condensed into 13 design solutions.
3	Manager (nurse, *n* = 1) and assistant manager (nurse, *n* = 1) from a home care team	Individual task to assess the 13 condensed solutions from workshop 2 in a 2×2 matrix (value and feasibility), followed by a shared assessment and discussion. (Define point of view).	The 13 design solutions were condensed into 6 design solutions.
4	Senior citizens (*n* = 2, age 74, 87); carers (*n* = 2, daughter-in-law, spouse; age 67, 84), health care professionals (nurse, *n* = 1; skilled health workers, *n* = 2); manager (*n* = 1, nurse), assistant manager (*n* = 1, nurse)	Individual task to assess the 6 condensed solutions from workshop 3 in a 2×2 matrix (value and feasibility), followed by individual dot voting and a shared assessment and discussion. Initial prototype building for the selected solution. (Define point of view, Ideate, Prototype)	Selection of “Professionals having sufficient time for their job / every senior citizen (prevents delays)” for the prototype building which led to 24 design solutions divided over 4 sub-themes: (1) less tasks for health care professionals; (2) less time thieves; (3) reorganization; and (4) improved health among HCP’s (less sick leave).
5	Head of the municipal home care services (nurse, *n* = 1), manager from the selected home care team (nurse, *n* = 1)	Email and telephone contact. Assessment (value and feasibility) of the 24 possible solutions from workshop 4 to select 5–7 possible solutions for workshop 6. (Prototype)	6 design solutions were selected.
6	Senior citizens (*n* = 4); carers (*n* = 2, daughter-in-law, spouse; age 67, 84), health care professionals (nurse, *n* = 1; skilled health workers, *n* = 2); manager (*n* = 1, nurse), assistant manager (*n* = 1, nurse)	Dot voting on the 6 solutions from workshop 5 on an A3 sheet of paper. Brainstorming (sticky notes) on three “visual roads” on an A3 sheet of paper, including: (a) how to achieve the selected solution; (b) possible obstacles; and (c) how to overcome these obstacles. Think-pair-share throughout the whole workshop. (Prototype)	Selection of “to support that senior citizens can manage themselves.” The “visual roads” led to 61 design solutions divided over 16 sub-themes, including assistive devices, competence in reablement, training/responsibility, collaboration, support from family/friends, etc.
7	Head of the municipal home care services (nurse, *n* = 1), manager from the selected home care team (nurse, *n* = 1)	Individual 2×2 matrix to assess the value and feasibility of design solutions from workshop 6; to be tested in the selected home care team (think-pair-share). (Prototype)	Selection of “to improve reablement and training” and “improve (reablement) competence among home care professionals.” Agreement on “job shadowing in the municipality’s reablement team” as main prototype.
Age-friendly mobility			
8	Municipal adviser (project leader for urban mobility)	Visual presentation (as workshop 1). Individual task to assess the 11 design solutions from workshop 1 (2×2 matrix; value and feasibility), followed by “share.” (Define point of view)	Selection of two design solutions: (1) on demand transport; and (2) design aspects with regard to the bus stop: kerb height, shelter possibility, distance between the kerb and the bus.
9	Senior citizens (*n* = 2, age 86, 90); senior citizens representative (*n* = 1, age 74); municipal advisers (*n* = 2); head of the municipal home care services (nurse, *n* = 1); advisers from the regional mobility provider (*n* = 2); bus driver from one of the local operators (*n* = 1)	Individual dot voting on the two selected design solutions of workshop 8. In three groups, the participants were asked to use artifacts (Playmobil figures, paper, glue, etc.) to build prototypes. Think-pair-share throughout the whole workshop. (Define point of view, Ideate, Prototype)	To improve “the distance between the kerb and the bus” was selected for the prototype building. Three groups developed prototypes.

a 10 participants joined multiple workshops.

b The design phases “define point of view,” “ideate” and “prototype” were carried out iteratively during the workshops.

### Data collection

**Figure 1:**
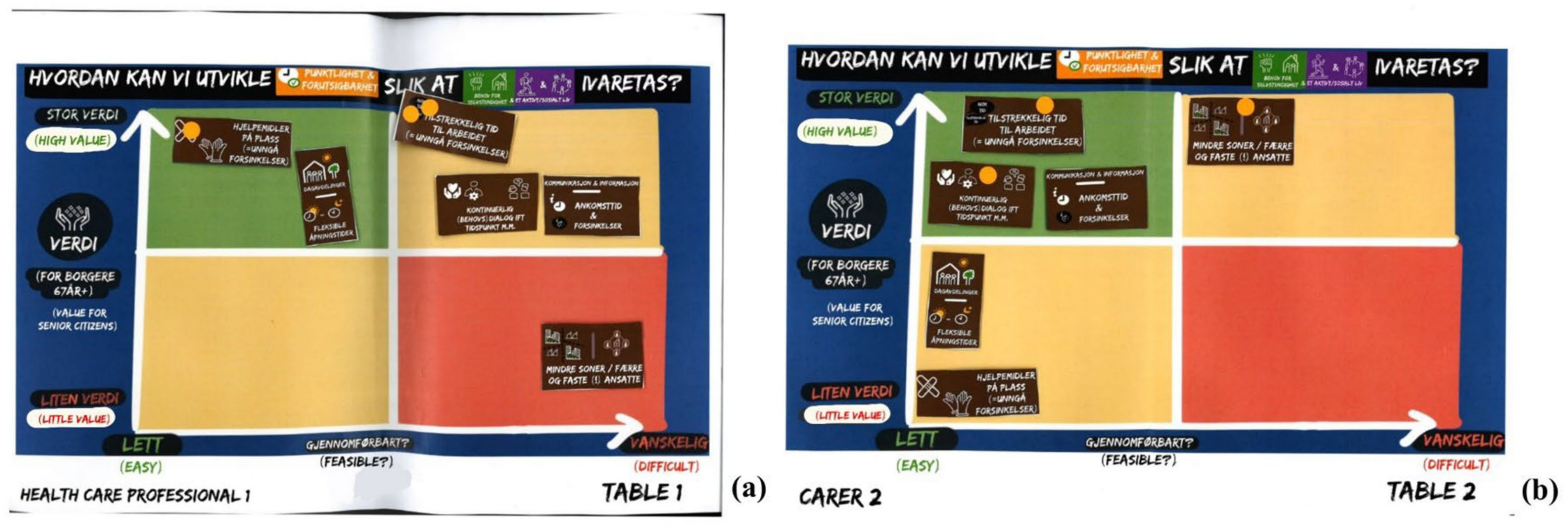
Examples of individual 2×2 matrices used in workshop 4 **(a,b)**, followed by individual dot voting **(a,b)**; conducted through think-pair-share. © 2023 Christophe Eward Kattouw.

**Figure 2:**
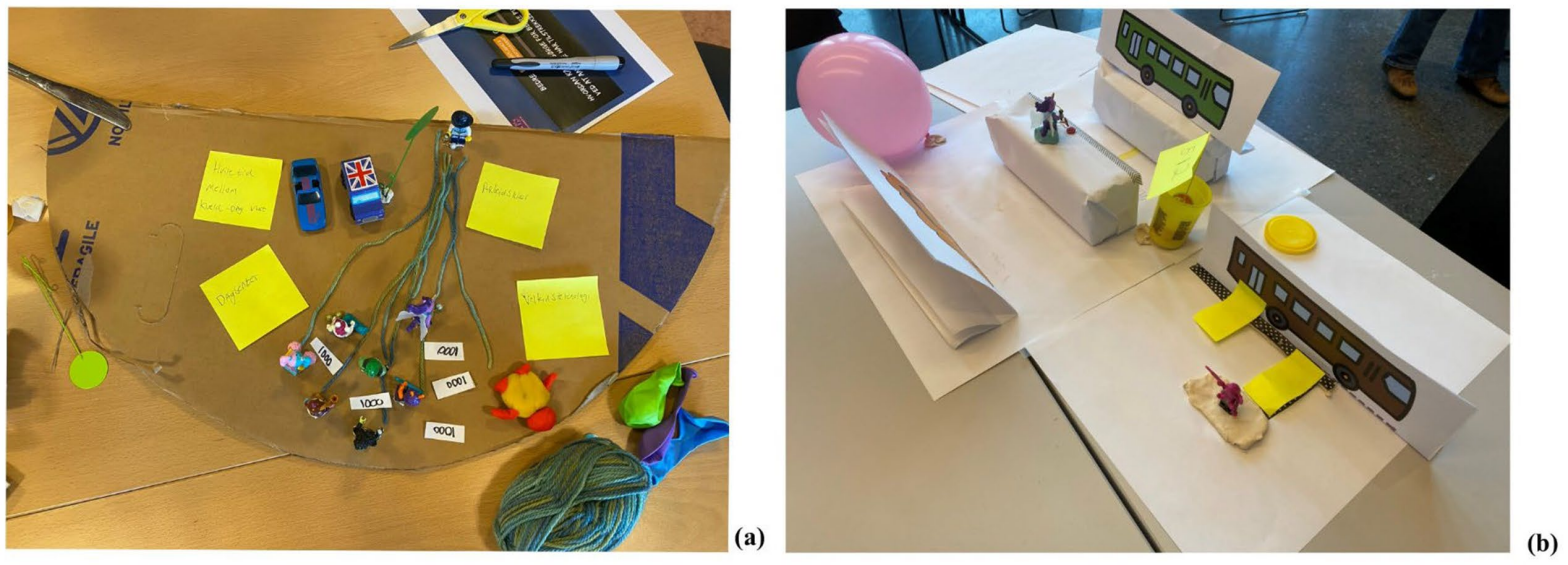
Examples of prototype building to improve timely home care provision in workshop 4 **(a)**, and age-friendly mobility in workshop 9 **(b)**. © 2023 Christophe Eward Kattouw.

Nine workshops were conducted (Table 1), three of which were planned and facilitated with the support of a professional designer. Participants were presented the study’s aim, and findings from previous workshops. During these workshops, they defined the point of view, focused on actionable problems for ideation and development of prototypes. Apart from workshops 7 and 9, all insights and potential design solutions were validated and assessed for their value and feasibility by the participants. It involved an iterative process of selecting prototypes for further development. Various design methods were used, including think-pair-share, 2×2-matrices, dot voting, lateral thinking tasks, negative brainstorming, bodystorming (simulation and role play), and prototype building (Table 1; Figures 1, 2) (25, 34, 37). At the end of every workshop, participants evaluated their workshop experience using think-pair-share, and shared feedback unsolicited during the workshops. All workshops were audio recorded (39–188 min) and transcribed verbatim. Notes and pictures were made by the first and last author, and the professional designer.

### Data analysis

An inductive thematic analysis (38) was conducted. All notes and quotes focusing on design solutions were extracted and transferred to Excel by the first author. Co-authors checked a random selection of quotes and found them to be accurate. The quotes were coded, and insights, sub-themes, and themes were discussed among the authors.

Table 2 provides an example of the inductive thematic analysis process. All three authors have considerable experience in clinical practice and qualitative data analysis. Involving all three authors, along with a professional designer, enabled multiple perspectives in the analysis and supported triangulation. The use of multiple data sources such as audio files, notes and pictures added to this (39). Triangulation, complemented by member checking, was also applied through multiple stakeholder groups assessing and validating the insights and potential design solutions for their value and feasibility during the workshops. To ensure sufficient information power (40), the initial number of workshops and participants was expanded to nine workshops involving 58 participants from diverse stakeholder groups (Table 1). Triangulation, member checking, and information power collectively strengthened the relevance and credibility of the analysis and findings (41).

**Table 2:** Example of the inductive thematic analysis process.

Transcript	Code	Sub-theme
Workshop age-friendly mobility		
More assistive devices are needed to enable them living at home. There must be good bus stops near apartment blocks, with good roads leading to the bus stops and there should be crosswalks to cross the road. A real-time app could inform about special needs such as wheelchair use and preparing the bus driver. Buses should have electric solutions with a ramp that automatically extends. […] We have challenged those working with urban planning to sit in a wheelchair or use a walker. And they gained some personal experience realizing that it wasn’t as accessible as they had thought.	Assistive devices	Enabling self-reliance
	Bus stops close to homes	Urban mobility
	Accessible roads to get to the bus stop	Urban mobility
	Bus stops equally distanced	Urban mobility
	Cross walks	Urban mobility
	App for special needs communication	Real-time communication
	Buses with electrical ramps	Urban mobility
	Challenge urban planners	Reshape patterns of thought
	Insights by using infrastructure	Reshape patterns of thought
Workshop timely home care provision		
Many tasks are scheduled at the same time. Spreading them out would make it more predictable for patients and professionals. Those handling schedules should be familiar with them and well-trained, to ensure predictability and that tasks are carried out correctly. There should be sufficient numbers of cars, to avoid driving people around due to a lack of vehicles. There should be sufficient staff. Reducing or avoiding evening-day shifts would reduce staff exhaustion and sick leave. Service users should have sufficient access to day centers. Assistive devices should be delivered swiftly. Technical devices could enable sending a message in case of delays.	Task distribution	Resource organization and flexible scheduling
	Familiar professionals	Collaboration and education
	Educated professionals	Collaboration and education
	Knowing what to do	Collaboration and education
	Sufficient cars	Urban mobility
	Sufficient staff	Resource organization and flexible scheduling
	Prevent sick leave	Resource organization and flexible scheduling
	Reduce evening-to day shifts	Resource organization and flexible scheduling
	Accessible day centers	Housing and buildings
	Swift delivery of assistive devices	Enabling self-reliance
	Digital communication of delays	Real-time communication

## Results

Design proposals were developed for the two selected improvement areas of timely home care provision and age-friendly mobility. They included prototypes and design principles for seven domains: (1) enabling self-reliance; (2) housing and buildings; (3) urban mobility; (3) collaboration and education; (5) real-time communication; (6) resource organization and flexible scheduling; and (7) methods and tools to reshape patterns of thought (Table 3). The design principles were centered around enabling sufficient time and resources, improving accessibility, and changing mental models.

**Table 3:** Prototypes ^a^ for timely home care provision and age-friendly mobility.

Domain	Both improvement areas	Improvement area 1: Timely home care provision	Improvement area 2: Age-friendly mobility
1. Enabling self-reliance	Comprehensive needs assessments Prioritization of reablement services and assistive devices Simplified application process for assistive devices, improved delivery time	Use of video conference and robots	
2. Housing and buildings	Housing cluster for senior citizens Combined multifunctional buildings, flexible opening hours, a variety of services		
3. Urban mobility	Sufficient transport options Improved parking options Home delivery by drones	Permission for professionals to use restricted roads	Kerbside stops (instead of bus bays) Spacious bus stops with shelters Spacious kneeling busesContrasting colors to improve visibility and accessibility
4. Collaboration and education	Facilitate multidisciplinary collaboration Continuous stakeholder involvement Support from family, carers and volunteers	Collaboration with schools and universities: students begin the day with practical training, followed by theory lessons Education to promote (reablement) competence and compassion Fast-track qualification Job shadowing in the municipality’s reablement team	Age-friendly mobility dashboard to provide information for infrastructural decision making Evaluation of infrastructural redesign projects
5. Real-time communication	Information on expected time of arrival Ability to communicate preferences and make reservations	Ability to communicate care preferences and make reservations	Ability to communicate need for assistance and use of mobility aids
6. Resource organization and flexible scheduling		Sufficient staff during peak hours Flexible rotation schedule Application that produces “digital home care visit lists” Digital application for home care professionals to book specific time frames Task distribution in teams Small teams	Sufficient bus lines, high frequency, extra time built into the schedule during midday period
7. Methods and tools to reshape patterns of thought	Design workshops Healthy aging and mobility campaigns Dialogues		Age-simulation suits Communication channels for improvements

a Empty cells under either “Timely home care provision” or “Age-friendly mobility” indicate that no prototypes were identified specifically for that domain, or that the prototypes applied to both areas and are therefore listed under “Both improvement areas.” Empty cells under “Both improvement areas” indicate that no shared prototypes were identified across the two domains.

The design proposals should enable senior citizens to live independently at home while staying active and socially engaged in their communities. They also have the potential to limit health care service needs, free up time and resources for service providers, and provide time margins to accommodate delays in service delivery. Meanwhile, the prototypes also require sufficient time and resources. Both the prototypes and their guiding principles demonstrate considerable interconnectedness and transferability within and across the two improvement areas (Table 3). Overall, the design proposals should improve predictability and adaptive capacity for both senior citizens and service providers.

### Enabling self-reliance

Reablement was suggested as a key strategy to promote and sustain senior citizens’ self-reliance, health, mobility, psychological safety, and engagement in social and physical activities. It relates to both improvement areas, and involves multidisciplinary meetings held in senior citizens’ homes, guided by user involvement and centered around the question “what matters to you?” A comprehensive needs assessment is carried out by a designated reablement team consisting of for example physiotherapists, occupational therapists, nurses, and nursing assistants. This team provides close follow-up, including home adaptations and or targeted training over a limited period of several weeks. While reablement can be of considerable value for senior citizens, it may also delay the need for services and free up time and resources for other service users.

Video conferences and assistive devices, including robots, could further improve timeliness, reduce the workload and thereby free up time for home care professionals, and support senior citizens’ mobility. Professionals who have easy access to assistive devices and necessary equipment can prevent service delays. Specialized home care professionals who navigate the complex bureaucratic systems may ease the application process for such technologies. A simplified ordering system and swift delivery, such as a “library service” model where citizens borrow devices using their library card, can further improve access. Although welfare technology might offer promising support, it should not replace human relationships and may not always suit senior citizens living with cognitive challenges.

### Housing and buildings

Housing clusters for senior citizens and multifunctional buildings near public transport can support senior citizens’ self-reliance, supporting them to remain socially engaged and physically active. Homes designed for life-long living which can accommodate assistive devices, can further enhance mobility and independence.

Participants recommended the development of multifunctional buildings inspired by dementia villages. These could offer a range of services, such as senior citizens centers with care provision, physical exercise facilities (including balance training), social meeting spaces, educational opportunities, demonstration rooms for assistive devices and welfare technologies, and grocery shopping. Such buildings could foster intergenerational interactions, limit the burden on health care professionals, and free up time and resources by enabling care delivered on-site rather than in individual homes. Accessibility could be further improved through flexible opening hours, while home delivery of groceries and meals could also promote senior citizens’ autonomy.

### Urban mobility

Ensuring accessible and sufficient transport options, alongside prioritized parking close to homes, grocery stores, and medical centers, could considerably enhance the everyday mobility of senior citizens and home care professionals. Readily available parking near key destinations not only makes daily errands such as grocery shopping more manageable for senior citizens but also frees up time for professionals by reducing their need to search for parking or walk long distances. This, in turn, enables them to spend more time for other purposes.

Additional mobility measures include a sufficient number of weather-resilient cars and reliable GPS systems to support efficient service delivery. Home care professionals should have access to restricted roads, and alternative transport options, such as mopeds to avoid traffic congestion, alongside weather-resistant clothing to prevent delays and reduce sick leave.

Drones can facilitate senior citizens’ access to essential goods (e.g., groceries and medicines) through home delivery and reduce wait times. They can also free up time for home care professionals by reducing the need for home visits. Moreover, drones equipped with cameras and verbal communication features can support medication adherence by reminding senior citizens to take their prescribed medicines correctly.

Replacing bus bays with curb bus stops may improve accessibility and mobility, especially for senior citizens using walkers or wheelchairs. Bus bays create unpredictable gaps between the bus and the kerb which impose considerable barriers. Compared to bus bays, kerbside stops enhance comfort for passengers and free up time for mobility providers, as they reduce the need for bus drivers to slow down three times before stopping, as well as making it easier for senior citizens to enter and exit taxis. Bus stops should be located near residential areas, be equipped with shelters, and designed for easy street crossing. To improve accessibility, bus stops should be spacious enough to accommodate bus ramps and have tactile paving with contrasting colors to assist visually impaired individuals. Incorporating ramps in bus stops can further enhance usability. Roads should be designed to accommodate larger busses, smaller buses for local neighborhoods, and bicycle lanes.

Buses are more accessible for senior citizens if they possess age-friendly features, like “kneeling” functions and ramps with spacious interiors for mobility aids and contrasting colors. Accessibility and mobility can also be improved through dial-a-ride services.

### Collaboration and education

Multidisciplinary collaboration is facilitated through regular meetings at municipal, county, and national level to access multiple perspectives. This helps to enhance the quality of decision-making. Regularly involving senior citizens in mapping their needs can help develop holistic plans and adjustments. Continuous stakeholder involvement, including senior citizens, carers, and GPs, may improve senior citizens’ health and self-reliance, and reduce care needs. Stakeholders can also learn from other fields and service providers such as kindergartens (e.g., working hours) and train providers (e.g., service and accessibility). Universal design benefits a wide range of other user groups such as passengers with prams. Continuous evaluation of redesign projects is important to adhere to universal design rules.

Support from family, carers, and volunteers could help senior citizens live at home, e.g., by providing groceries and transport. Volunteers, including pensioners, can assist during peak hours of home care and assist senior citizens to enter or exit buses. People re-entering the workforce may serve as hosts and gain job experience.

Collaboration with schools and universities may improve timely home care provision through student practice. Students can provide care to senior citizens in the morning and then share their experiences during classroom sessions. This not only supports senior citizens and frees up time for home care professionals but also offers students valuable hands-on learning.

Education may promote competence and compassion, and the provision of education in multifunctional buildings should be accessible to all, including senior citizens, volunteers, students, home care professionals, and bus drivers. Education may enhance senior citizens’ competence in using assistive devices, including smartphones and other electronic devices. Education may also improve predictable, holistic and compassionate home care by professionals seeing the person rather than the task. This requires physical and mental presence, good communication skills, and speaking the native language. It may also enhance empathy and competence for age-friendly mobility, such as how kerb gaps affect seniors’ mobility. Home care professionals may develop an increased sense of responsibility and see the value of their attendance at work, to be accessible on the phone, and to inform senior citizens in case of delays. They may gain a better understanding of the consequences of non-attendance, extended breaks, and private errands. Unskilled workers should quickly obtain necessary qualifications to serve as a resource.

Professional development can free up time and enhance adaptive capacity, enabling professionals to manage various situations independently. Stakeholders should learn more about specific health challenges, such as living with dementia, and evidence-based skills to develop better solutions and to work time effective. Competence in reablement is important to promote seniors’ self-reliance. Observing and learning through job shadowing in reablement teams was suggested as a key solution and can promote home care professionals’ competence in reablement. Developing skills in assessing and using assistive and digital devices can further promote senior citizens’ self-reliance.

### Real-time communication

Real-time estimated time of arrival and delay communication devices are common among mobility providers to enhance predictability for passengers. To ensure these devices are accessible to senior citizens, they must be designed with clear visibility, strong audibility, and user-friendly interfaces. This applies to bus stops, in-bus screens, and smartphones. Similar technology should be available for home care services. Devices that provide real-time updates on arrival times, reasons for delay, and details about the professionals visiting, can improve predictability and reduce the need for telephone calls, freeing up time for care providers. However, certain devices may not suit all senior citizens, e.g., those living with cognitive and or visual impairments. Digital booking systems and communication of preferences can benefit both home care- and mobility services, prepare bus drivers for passengers with special needs, and inform home care professionals about senior citizen’s time preferences, enhancing predictability for professionals and reduce reliance on phone communication. Home care professionals should communicate honestly about delays and negotiate arrival times respectfully.

### Resource organization and flexible scheduling

A flexible organization and distribution of care tasks through a flexible rotation schedule can better accommodate the evolving care needs of senior citizens. This could be implemented by special shifts or extended (12–14-h) shifts, especially during home care’s peak hours (8-10 a.m. and 8-10 p.m.). However, a holistic approach is essential. This includes considering home care professionals’ health, working hours preferences, and the need for sufficient rest between shifts. This ensures sufficient time and resources, also improving professionals’ health and attendance. Fewer visits, allocated with more time, can enhance timeliness and support dialogue with senior citizens, to better assess their needs and develop solutions according to what matters to them. The reablement process requires more time to enable senior citizens to gradually regain independence, rather than having professionals take over and execute care tasks for them.

The development of digital applications may facilitate scheduling processes, improve access to valuable information, and free up time and resources. A single application must provide digital home care visits lists based on staff availability, professional competencies, senior citizens’ care needs and time preferences, geographic location, and travel time. It should also address the need for a limited number of professionals visiting the same senior citizen over time. The app should integrate another system providing staff needs at certain time frames based on the number of senior citizens, their care needs, and time preferences throughout the week. This information may improve predictability for home care providers, and allows professionals, students and volunteers to confirm their availability and digitally book or accept shifts.

Improved transportation access, such as dial-a-ride services using minibusses requires sufficient staffing. Higher frequency bus lines can eliminate the need for timetables, making public transport more accessible to seniors. Age-friendly mobility can also be enhanced through cheaper or free public transport. Fewer transfers and longer routes with built-in buffer time during midday hours (10 a.m.–2 p.m.) can improve accessibility, though longer bus lines may cause delays. However, mobility providers must balance interests and needs for punctuality and predictability.

To further free up time for home care professionals, an alarm team could manage and respond to alerts, phone calls, and e-messages from the GPs or hospitals. Rapid response teams and helping to bed teams may improve timeliness and free up time but may reduce continuity of care and familiarity for senior citizens.

Smaller home care teams assigned to smaller districts can enhance timeliness, predictability, and relationship building. Professionals in these teams become familiar with the area and with senior citizens’ routines and preferences, enabling more personalized and time effective care. Care provision by one professional instead of multiple staff across different times can also free up time. Expanding user-controlled personal assistance can improve familiarity, involvement, and better adapted solutions for senior citizens. However, smaller teams may be more vulnerable during periods of sick leave and holidays.

### Methods and tools to reshape patterns of thought

Design workshops involving multiple stakeholders may reshape patterns of thought by facilitating the exchange of different perspectives. They can provide mutual insights into stakeholders’ needs, opportunities, and working routines of home care professionals and bus drivers. According to the participants, this can lead to eye-openers, enhance creativity and innovative thinking.

Wearing age-simulation suits and using assistive devices such as walkers and wheelchair can add to this. Stakeholders such as home care professionals, bus drivers, mobility providers, architects, and builders, may enhance their empathy, competence and mutual understanding of the value of age-friendly mobility. Bus drivers may become more aware of the consequences of how the gap between the bus and kerb may affect senior citizens’ mobility, prompting bus drivers to change their driving behavior and approach to the bus stops.

Through meaningful dialogues with senior citizens, home care professionals may set realistic expectations about arrival times and map specific mobility needs. Aging and mobility campaigns may help shift attitudes among senior citizens, and help to highlight the benefits of physical activity, balance training, and future planning. Moving into an apartment or senior citizen housing cluster earlier in life can promote independence, social engagement, and delay the need for services.

Mobility campaigns may encourage peer support among bus passengers and help change attitudes towards public transport. More senior citizens may take the bus, and reduce the need for private transport and the stigma due to losing their driver’s license. However, changing long-standing habits and attitudes often becomes challenging with age.

Service improvements to promote the use of age-friendly mobility should be communicated to senior citizens through social media (e.g., Facebook), TV commercials, local newspapers and (pensioners’) magazines, information at the bus stop and senior citizen centers, as well as through verbal and written information provided by home care professionals.

### Design principles

Enabling sufficient time and resources, improving accessibility, and changing mental models emerged as design principles for the two selected improvement areas. Although the prototypes for the seven domains may free up time and resources, improve accessibility and enable mental model change, their successful implementation also depends on the availability of sufficient time and resources.

Enabling sufficient time means allocating adequate time for both home care and public transportation providers to effectively serve senior citizens. It also involves building in time margins to accommodate unpredictable delays in service delivery. Such delays may occur due to, e.g., senior citizens’ varying needs and health condition, fluctuations in staffing due to sick leave, holidays, and weekends, and differences in infrastructure, equipment, and assistive devices. Weather conditions can also affect mobility. Sufficient resources require adequate financial resources, equipment, assistive devices, and sufficient permanent staff who are fairly compensated. Improving accessibility encompasses the ability of senior citizens to be mobile within and outside the homes. It also includes access to home care and transportation services; supportive infrastructure (e.g., roads, parking places), assistive devices and equipment such as mobility means for both senior citizens and home care- and mobility providers. Accessibility also involves availability of real-time information of value for both senior citizens, home care and transportation providers, and policy- and decision makers. When this information provides insights into what, when, how, and by whom, it enhances predictability and allows for better adaptation to continuous changing needs and preferences. Changing mental models involves a reshaping of stakeholders’ thought patterns, attitudes and understanding the value and urgency of transformation, especially among policy- and decision makers. Mental model change aligns closely with the objectives of education.

Sufficient resources make it possible to ensure sufficient time and improved accessibility. In turn, sufficient time and improved accessibility can enable senior citizens to board and disembark the bus, and to sit down before the bus starts moving again. Sufficient time enables assistance from others, including the use of assistive devices such as ramps. Sufficient time and resources enable service providers to do a proper job in direct contact with senior citizens as well for, e.g., coordination and administrative tasks. The ability to do a good job and having sufficient time to rest, may promote professionals’ health and attendance at work, and thereby support sufficient time and resources. Sufficient time is essential for good communication and dialogues with senior citizens, and enables home care professionals to sit down, to be relaxed and to do proper needs assessments. It also allows professionals to reflect on and improve their practice, and to make necessary preparations. Moreover, sufficient time enables home care professionals to work with their hands behind their back as part of the reablement process. Senior citizens are then enabled to execute care tasks themselves instead of home care professionals taking over due to time constraints. As such, enabling senior citizens’ self-reliance through reablement and assistive devices can free up time and resources.

Sufficient time and resources are essential for changing mental models. Attitude changes and development of holistic perspectives in a context of continuous improvement require investment in time and education. Mental model change among politicians and decision makers makes them more likely to value and prioritize aging and health promotion, e.g., through increased financial investments. According to the participants, the budget for home care and age-friendly mobility should be increased. Not only may this improve predictability and adaptive capacity, but it may also result in long-term savings by freeing up time and resources.

## Discussion

This study offers a unique contribution in providing design proposals aimed at closing gaps between the existing and the preferred service ecosystems for senior citizens living at home. The proposals targeting timely home care provision and age-friendly mobility include prototypes and design principles with considerable interconnectedness and transferability. The findings have implications for service provision and advance current understandings of service ecosystem transformation, systems thinking, and service design methods.

### Expanding upon existing design solutions

Several prototypes for the seven domains of timely home care provision and age-friendly mobility have been described in previous studies including for example reablement, improved parking options, home delivery by drones, support from family, carers and volunteers, and real-time communication (3, 42–49). However, many of these prototypes either focus on improving age-friendly mobility, have low fidelity, or address fewer domains. None of the prototypes presented in the literature were considered to represent solutions for timely home care provision. Additionally, prototypes such as kerbside stops, collaboration with schools and universities, and job shadowing within reablement teams, were unique for this study.

Research on service unpredictability, waiting times and allocated time in home care services is scarce. Existing studies suggest that senior citizens and home care professionals continuously adapt to time constraints, highlighting a need for improvement (50–52). Our study addresses this need providing various and detailed examples of unpredictability, together with stakeholders’ adaptations to the service provision. This aligns with the service-dominant logic and underscores the understanding of the service ecosystem as a complex adaptive system (10, 53).

Previous research suggests that predictability and adaptive capacity in service ecosystems could be enhanced through sufficient information and knowledge (27, 53), also confirmed by our study and expanded with the importance of real-time communication, accessibility, and margins for delays.

### Systems thinking and service ecosystem transformation

In line with idealized design and systems thinking (21, 27, 54, 55), our study suggests that an integrated implementation of the design proposals may offer more value for senior citizens and service providers than implementing single prototypes and design principles. For example, the prototype “real-time communication on bus arrival times” only holds value for senior citizens if they also have access to the bus itself. The same applies to assistive devices, which require housing and buses that provide sufficient space. Interconnectedness and transferability of the design proposals was also demonstrated through the applicability of prototypes and design principles across both improvement areas.

The development of design principles is still in its early stages and is therefore rarely described in the service literature. Design principles are “high-level” guidelines, including empirical insights and situated experience found inductively, that form the basis for and guide solutions of broader clusters of gaps (33, 56). In this context, the four design principles which emerged in our study may support the transformation of the service ecosystem for senior citizens living at home.

While various previous studies have focused on improving efficiency in health care systems, such as minimizing the use of time and resources (57–59), our findings suggest that allocating sufficient time and resources may support the overall effectiveness of the service ecosystem for senior citizens living at home. A Dutch study demonstrated that extending GP consultation times resulted in fewer referrals to specialized care (60). Similarly, a study in a Norwegian nursing home documented that investing resources in staffing led to improved care quality, reduced sick leave, and positive financial outcomes (61).

Identifying leverage points is considered as one of the key foundations for systems thinking and system improvement. Leverage points are places in complex systems where a relatively small change may lead to a large shift in the system’s behavior, considered as “points of power” (62–65). Mental model change was identified as a key leverage point in our study. Moreover, the findings suggest that also the other design principles could be considered as key leverage points for transforming the existing service ecosystem although they are not described as such in the service literature. The design prototypes (Table 3) also indicate that seemingly “simple” measures can be impactful for multiple stakeholders. For example, improving accessibility by developing kerbside stops instead of bus bays, or fostering collaboration with schools and universities to achieve age-friendly mobility and timely home care provision.

### Workshop design methods

The overall feedback during design workshops was positive regarding the development of design proposals, but also in achieving new and mutual insights and perspectives across the stakeholder groups.

Established design methods such as 2×2 matrices and dot voting (25, 37) were modified for the design workshops of this study. The 2×2 matrices were developed for individual use and after individual dot voting, participants were asked to compare and discuss their individual assessments in pairs, before sharing in a larger group. The intention was to support reflective processes through active participation and psychological safety. This appeared to be a useful approach for all design workshops in which participants collaborated and helped each other in pairs and groups. An even distribution of different stakeholder groups further supported the participation of senior citizens, who could ask their “neighbor” for clarification and help.

Framing of workshop methods and associated terminology was useful. For example, presenting “role play” as a “simulation” helped to ensure participation from certain stakeholder groups such as managers.

### Strengths and limitations

The findings of our study may expand the existing service literature by advancing current understandings of systems thinking, service ecosystem transformation, and service design methods.

Although a broad variety of stakeholder groups participated in the design workshops, other relevant stakeholder groups (e.g., politicians) could have been useful participants. Furthermore, perspectives, assessments and potential design solutions could have been retrieved from senior citizens through individual interviews in the senior citizens’ homes. However, despite some recruitment challenges with senior citizens, most of the attending senior citizens in the design workshops provided useful insights and participated actively.

Although the development of prototypes and design principles is an essential step for the transformation of service ecosystems, they must be tested and refined in practice and more research is needed to assess their value. Nevertheless, the solutions on for example reablement, the implementation of kerbside stops, and enabling margins for service delays would represent a viable foundation for implementation.

The data were collected in one Norwegian municipality thus representing one specific setting. The design proposals may nevertheless have value for other service ecosystems in other settings based on the detailed accounts of the design process and its context.

### Implications

The current study has multiple implications for practice. The developed proposals for timely home care provision and age-friendly mobility have the potential to improve predictability and accessibility and to support senior citizens in being self-reliant, social, and active in the local community. They also have the potential to provide sufficient time and resources for senior citizens, carers and service providers. To attain this, housing corporations and welfare organizations should invest in the development of age-friendly housing and buildings. Schools, universities and voluntary organizations should facilitate collaboration with home care and transportation providers. Politicians and decision-makers should emphasize collaboration and stakeholder involvement in the development of the preferred service ecosystem and increase competence among all relevant stakeholder groups, including urban architects and home care and transportation providers. They should safeguard sufficient time for service provision, increase the budgets for home care and transportation, and improve accessibility. Design methods for the involvement of senior citizens need further development.

Implications for research is related to the interconnection between timeliness, margins for delays, and competence on the one hand, and predictability and adaptive capacity on the other hand. Another focus area is how different stakeholders manage unpredictability in service provision, as well as implementation research to assess how sufficient time, resources, and accessibility, could serve as design principles for the preferred service ecosystem for senior citizens living at home.

## Conclusion

This study contributed with design proposals across seven domains related to timely home care provision and age-friendly mobility, demonstrating considerable interconnectedness and transferability between the domains. The design proposals included novel prototypes and design principles with the potential to support senior citizens’ self-reliance, free up time and resources for service providers, and provide time margins to accommodate delays in service delivery. Overall, the design proposals may improve predictability and adaptive capacity for both senior citizens and service providers. The findings have implications for service provision and advance systems thinking, service ecosystem transformation, and service design methods.

## Data Availability

The raw data supporting the conclusions of this article will be made available by the authors, without undue reservation.
